# The Effect of Esophagogastroduodenoscopy on Intraocular Pressure

**DOI:** 10.3390/jcm13051224

**Published:** 2024-02-21

**Authors:** Maddalena De Bernardo, Antonella Santonicola, Marco Gioia, Livio Vitiello, Ferdinando Cione, Sergio Pagliarulo, Paola Iovino, Nicola Rosa

**Affiliations:** 1 Eye Unit, Department of Medicine, Surgery and Dentistry “Scuola Medica Salernitana”, University of Salerno, Baronissi, 84081 Salerno, Italy; mdebernardo@unisa.it (M.D.B.); mgioia@unisa.it (M.G.); livio.vitiello@gmail.com (L.V.); fcione@unisa.it (F.C.); sergiopagliarulo88@gmail.com (S.P.); nrosa@unisa.it (N.R.); 2 Gastroenterology Unit, Department of Medicine, Surgery and Dentistry “Scuola Medica Salernitana”, University of Salerno, Baronissi, 84081 Salerno, Italy; piovino@unisa.it

**Keywords:** intraocular pressure, esophagogastroduodenoscopy, supine position, lateral decubitus position, sedation, intra-abdominal insufflation

## Abstract

Background: Esophagogastroduodenoscopy (EGD) is an endoscopic examination of the upper gastrointestinal tract that requires insufflation with gas, leading to intra-abdominal hypertension (IAH). There is evidence suggesting that IAH positively correlates with intracranial pressure (ICP) and possibly with intraocular pressure (IOP). The aim of this study was to examine the effect of a routine screening EGD on the IOP. Methods: In this observational study, 25 patients were recruited; 15 males with a mean age of 50 ± 18 years and 10 females with a mean age of 45 ± 14 years. EGD was conducted under sedation in 21 subjects. Both eyes’ IOP measurements were performed using Tonopen Avia in the sitting and left lateral decubitus positions before sedation and the start of EGD, and subsequently in the left lateral decubitus position when the endoscope reached the duodenum (D2) and at the end of the procedure. The final measurement was performed in the sitting position 10 min after the end of the procedure. Results: The mean IOP in the sitting position was 15.16 ± 2.27 mmHg, and in the left lateral decubitus position, 15.68 ± 2.82 mmHg. When the gastroscope entered the D2, it was 21.84 ± 6.55 mmHg, at the end of the procedure, 15.80 ± 3.25 mmHg, and 10 min later, 13.12 ± 3.63 mmHg. There was a statistically significant IOP increase when the gastroscope entered the duodenum (*p* < 0.01). At the end of the gastroscopy, the IOP significantly decreased compared to the one registered when the gastroscope entered the D2 (*p* < 0.001) and it became similar to the values measured before the EGD, in the same left lateral decubitus position (*p* > 0.05). Conclusion: Significant changes in IOP were observed during the EGD. IOP fluctuations during EGD should be taken into account, especially in patients that need repeated EGDs during their life or in patients with glaucoma. Further studies are needed to better understand the short-effect and long-effect influence of an IOP increase in these patients.

## 1. Introduction

Intraocular pressure (IOP) is still a key parameter in the evaluation and management of glaucomatous patients, as well as the only adjustable risk factor linked to glaucoma progression. IOP could be affected by several factors. Among the factors, body position, diurnal variations, heart rate, and medications seem to play a role [1].

Endoscopic procedures continue to play a pivotal role in the diagnosis and treatment of upper and lower gastrointestinal (GI) disorders. Recently, Kent et al. observed a statistically significant IOP decrease at the end of colonoscopy [2]. In fact, the endoscopic examination of the colon requires air insufflation to adequately visualize the colonic mucosal and detect any lesions. The air insufflation of the colon is associated with the increased intra-abdominal pressure that could influence both the intracranial pressure and the IOP [3,4,5,6,7,8,9]. Esophagogastroduodenoscopy (EGD) is a high-frequently performed endoscopic procedure that examines the esophagus, stomach, and the first part of the small bowel (duodenum). The procedure is often performed under sedation with a fiber optic camera on a flexible tube and it is used to investigate, diagnose, and treat several diseases [10]. In recent years, a standard protocol for EGD has been highly recommended to ensure a good-quality endoscopy, with the detection of all the precancerous lesions and early cancers in the upper GI tract [11,12]. In particular, the importance of a complete mucosal inspection, achieved by a combination of adequate air insufflation, aspiration, and the use of mucosal cleansing techniques has been underlined. In addition, a high-quality EGD requires time; the whole procedure should take on average 7 min, but the duration can be prolonged in the case of high-risk and surveillance procedures such as Barrett’s esophagus or gastric atrophy surveillance [11].

The air insufflation for the luminal distension during EGD might lead to the increase in intra-abdominal pressure that possibly influences the IOP. However, to date, there are no studies investigating this topic.

The aim of the present study is to verify if IOP changes occur during routine EGD.

## 2. Materials and Methods

This is an observational prospective study. The study protocol was approved by the institutional ethics committee (Cometico Campania Sud prot. Number 16544). A written informed consent was obtained from each subject, after the nature and the intent of the study had been fully explained. Recruitment was performed at a single investigational site between February and July 2021. The study included subjects scheduled to undergo EGD for the diagnosis, screening, and follow-up of main gastrointestinal diseases, performed either under conscious sedation or without it. Inclusion criteria were subjects aged 18 years or older, with no history of glaucoma, no significant ocular or orbital pathologies, no specific abnormalities that may affect the IOP measurements’ performance or reliability, no history of severe ocular trauma or surgery, including ocular laser procedures [13], and no chronic ophthalmic medication use.

IOP was measured using Tonopen AVIA^®^ (TPA) (Reichert Ophthalmic Instruments, Depew, NY, USA), previously administering lidocaine 40 mg/mL eye drops. The IOP measurement was performed by an ophthalmologist who was familiar with the procedure. To measure the IOP, TPA utilizes the same GAT physical principle, on a smaller applanated area. In fact, the transducer tip, protected by a single-use latex tip cover before each measurement, has a 1.0 mm diameter. The mean IOP readings were automatically averaged by the instrument, when ten valid readings were obtained, by lightly touching the central cornea. The measurement was shown on the liquid crystal display, which is situated on the side of the device, and together it displayed the “statistical confidence indicator”, indicating that the standard deviation of the valid measurements is 5% or less of the number shown. The higher the value, the more reliable the measurement is. Only values higher than 90 were accepted. IOP measurements were performed in both eyes in the sitting and left lateral decubitus positions before sedation and the start of EGD, and subsequently in the left lateral decubitus position when the endoscope reached the duodenum (D2) and at the end of the procedure. The final measurement was performed in the sitting position 10 min after the end of the procedure. All measurements were made with precision so that the average of the 10 measurements made by the tonometer achieved 95% reliability. The mean IOP of both eyes for each time point of the procedure was computed and compared.

Conscious sedation with intravenous midazolam (5 mg) and eventually fentanyl (0.05 mg) was proposed to all participants, in order to avoid the discomfort of the procedure, even if maintaining autonomous respiratory function.

### Statistical Analysis

The sample size was calculated as follows: α error was set at 0.05, 1-β error was set at 0.80, and effect size was set at 0.8, and a non-central parameter λ of 16, critical F of 2.87, numerator df 4, denominator df 20, total simple size 25, and power of 0.82 were obtained.

Kolmogorov–Smirnov test was performed to evaluate the normal distribution of data and ANOVA post-hoc test with Bonferroni correction and Friedman test were used to compare the IOP measurements within the 4 time points during the examination; *p* < 0.05 was considered significant.

All data were analyzed with SPSS Software (IBM SPSS Statistics version 25).

## 3. Results

Fifteen males (60%) with a mean age of 50 ± 18 years (range 24–78) and ten females (40%) with a mean age of 45 ± 14 years (range 21–67) and mean weight of 73.76 ± 14.43 kg (range 50–117) were evaluated. Intravenous midazolam (5 mg) for conscious sedation during the EGD was performed in 13 patients, whereas 8 patients were sedated with midazolam (5 mg) and fentanyl (0.05 mg) by an experienced gastroenterologist. Four subjects refused sedation.

In all patients, the mean IOP in the sitting position was 15.16 ± 2.27 mmHg, and in the left lateral decubitus position, 15.68 ± 2.82 mmHg; when the gastroscope reached the second part of the duodenum (D2), the IOP was 21.84 ± 6.55 mmHg and, immediately after the gastroscope was removed (end of the procedure left lateral decubitus position), the IOP was 15.80 ± 3.25 mmHg (Table 1). The IOP in the sitting position, 10 min after the EGD, was 13.12 ± 3.63 mmHg. When the gastroscope entered the D2, a statistically significant IOP increase (*p* < 0.01) was observed. The IOP values at the end of the gastroscopy significantly decreased (*p* < 0.001), becoming similar to those measured before the EGD, in the same left lateral decubitus position. A further decrease was observed in the IOP values 10 min after the EGD, which became similar to those measured before the EGD, in the same sitting position (Table 2).

In the 21 patients under sedation, the mean IOP in the sitting position was 14.81 ± 2.25 mmHg, and in the left lateral decubitus position, 15.57 ± 2.60 mmHg; when the gastroscope reached the second part of the duodenum (D2), it was 21.88 ± 6.60 mmHg, and immediately after the gastroscope was removed (end of the procedure in left lateral decubitus position), the IOP was 16.40 ± 3.72 mmHg, while 10 min later, it was 12.98 ± 3.05 mmHg. When the gastroscope entered the duodenum (D2), a statistically significant IOP increase (*p* < 0.01) was observed.

Sedated patients were further divided into two groups, one sedated with midazolam alone, the other with a mixture of midazolam and fentanyl, to detect the eventual influence of different sedations on the IOP. A similar tendency in IOP variations was observed, without statistically significant differences among the two groups in all the examined positions (*p* > 0.05).

In the four patients that refused sedation, the mean IOP was 17.12 ± 2.49 mmHg, and in the left lateral decubitus position, 16.25 ± 1.55 mmHg; when the gastroscope reached the second part of the duodenum (D2), it was 20.00 ± 2.48 mmHg, and immediately after the gastroscope was removed (end of the procedure in left lateral decubitus position), the IOP was 16.37 ± 1.03 mmHg, while 10 min later, it was 15.25 ± 3.48 mmHg. In this group, a similar trend in IOP variations was observed, but it did not reach a statistical significance (*p* = 0.358).

## 4. Discussion

The IOP increase is considered the main cause of optic nerve damage as well as the only modifiable known risk factor in glaucoma patients. An acute IOP increase does not cause chronic glaucoma, but a strict IOP control is imperative for patients with a known diagnosis of glaucoma, where a transient IOP change can also be a determinant.

Previous studies showed a relation between intra-abdominal pressure, intracranial pressure, and IOP, and some authors evaluated the IOP changes after laparoscopy [6] and colonoscopy [2].

Grosso et al. [6] revealed a mean IOP increase of 4 mmHg after pneumoperitoneum induction, with 58.6% of the patients showing an IOP increase of 5 mmHg or more. Moreover, the Trendelenburg position during surgery exhibited both an IOP increase and a great percentage of cases with an IOP increase of 5 mmHg or more.

Ackerman et al. [6] reviewed the literature concerning the impact of a steep Trendelenburg position during robot-assisted laparoscopic radical prostatectomy on intraocular pressure.

Other studies [14,15,16,17,18] showed a direct association between a steep Trendelenburg position and increased IOP.

Yoo et al. showed low intra-abdominal pressure resulting in the significant attenuation of the IOP increase in 67 patients undergoing robotic-assisted laparoscopic radical prostatectomy, divided into a moderate neuromuscular blockade group and deep neuromuscular blockade [14].

Hoshikawa et al. demonstrated that the IOP increased in a time-dependent fashion in 31 anesthetized patients undergoing robotic-assisted radical prostatectomy in a steep Trendelenburg position [15].

Mondzelewski et al. reported a significant elevation in IOP in 18 patients during robotic-assisted laparoscopy in a steep Trendelenburg position [16].

Both Kim et al. and Raz et al. found an IOP increase in patients during robotic-assisted radical prostatectomy in a steep Trendelenburg position, which was attenuated by the continuous infusion of dexmedetomidine or a modified Trendelenburg position [17,18].

Ece et al. [4] showed that 12 mmHg or more pressure after pneumoperitoneum induction led to a significant IOP rise, with an average of 8.5 ± 3.4 mmHg. Moreover, they postulated a correlation between the IOP elevation and the intracranial pressure increase, caused by the intra-abdominal pressure elevation.

Kent et al. [2] measured the right eye IOP in a left decubitus position in 23 healthy adults undergoing routine colonoscopy. The authors demonstrated that the IOP did not increase during colonoscopy, although patients were in the left decubitus position. On the contrary, they revealed that the IOP progressively decreased during the progression of the endoscope with a maximal decrease when it reached the cecum.

However, to the best of our knowledge, no previous studies have investigated IOP changes during EGD. We found that the IOP increased when the endoscope passed through the pylorus and entered the duodenum. The suggested mechanism underlying these results is unknown. However, an increase in intra-abdominal pressure during EGD has been detected in an animal study [3]. When intra-abdominal pressure increases, an impaired venous drainage of the lumbar venous plexus is detected, eventually leading to the increase in intracranial pressure [19]. Moreover, the intracranial pressure elevation has been suggested to be related to the IOP increase. Specifically, the IOP rise could be due to the elevation in ophthalmic venous pressure, which would be directly transmitted to the ocular fluid [7,9,20].

In this study, the EGD was performed in 21/25 subjects under conscious sedation. Previous papers showed that midazolam does not modify IOP, whereas fentanyl induces an IOP decrease during its administration [21,22]. Therefore, we hypothesize that the IOP increase during the EGD was not related to drug administration. Moreover, our results highlighted an IOP increase mainly in the group of patients that underwent sedation, without significant differences between patients who received midazolam and those who received midazolam and fentanyl. A similar trend in IOP variations was also observed in the small group of patients who did not undergo any conscious sedation, but it did not reach a statistical significance, possibly due to the small number of patients. Further studies in a larger group of patients without sedation are needed to better clarify the role of anesthesia in the IOP changes.

The left lateral decubitus position could also contribute to explaining our findings. In fact, previous studies demonstrated that the right or left lateral decubitus position might be associated with a small IOP increase in the lower side, compared to the sitting position [23]. However, in our study, we found a significant IOP elevation in both eyes, when the gastroscope reached the second part of the duodenum (D2). So, we can speculate that other factors, other than the body position, might play a role.

The elevation in IOP is considered the major risk factor for glaucoma; in addition, it has been related to an increased risk of several ophthalmic conditions such as retinal vein occlusion and anterior ischemic optic neuropathy. Moreover, the failure to achieve the target IOP has been associated with a more rapid visual field worsening in patients with glaucoma [24].

In the last few decades, endoscopic procedures have grown up worldwide, becoming an important diagnostic and therapeutic tool in daily clinical practice. The introduction of image-enhanced endoscopy and magnifying endoscopy has improved the possibility to also detect pre-neoplastic and neoplastic lesions of the upper GI tract during gastroscopy [25]. Moreover, in recent years, scientific societies have highly stressed the importance of “quality gastroscopy”, performed according to a standardized protocol in order to maximize the detection of all the precancerous lesions and early cancers in the upper GI tract [11]. A high-quality EGD requires adequate air insufflation to better visualize the GI lumen and implies the use of several pieces of technical equipment to obtain the meticulous inspection of the mucosa and eventually the acquisition of histological samples [11].

Despite these undoubted advantages, we might take into account that the IOP increases during these endoscopic procedures and it could be a potential risk factor for optic nerve damage in glaucoma patients. It is important to underline that a transient IOP elevation during diagnostic procedures does not necessarily induce glaucoma, but care should be taken in patients with a previous glaucoma diagnosis.

One of the main limitations of this study was the use of TPA instead of GAT. In fact, GAT is based on the so-called Imbert–Fick law and is the gold standard for IOP measurement [26]. However, several factors, such as the central corneal thickness (CCT), curvature (Km), and structure, can influence its accuracy. In addition, taking into account the changes in the body position of the patients during the present study, GAT was unable to be properly managed. Furthermore, TPA has been used in similar studies where a handheld tonometer was required [27], showing comparable results.

Another limitation is the different sedating procedure among the patients, as a small group refused any kind of sedation, whereas most of the participants received midazolam alone or in combination with fentanyl. Although a similar trend in IOP variations was observed, further studies on a larger population could better clarify the role of sedation on IOP.

## 5. Conclusions

In conclusion, the IOP increase is considered the main cause of optic nerve damage in glaucoma patients, and IOP fluctuations during endoscopic procedures should be taken into account, especially, in patients that need repeated gastroscope procedures during their life or in patients with glaucoma. This is particularly important if glaucoma is in an advanced state where IOP spikes could induce severe damage.

In this paper, we detected significant changes in IOP changes during routine EGD. However, further studies are needed to better understand the short-effect and long-effect influence of the IOP increase in these patients and to suggest in future a possible preventive therapy.

## Figures and Tables

**Table 1:** Intraocular pressure evaluation (in mmHg) during different procedure times with Tonopen Avia.

	Sitting Position Pre-EGD	Left Lateral Decubitus Position Pre-EGD	Second Part of Duodenum in Left Lateral Decubitus Position	End of Procedure in Left Lateral Decubitus Position	Sitting Position 10 min after EGD
**Mean**	15.18	15.68	21.58	16.40	13.34
**SD**	2.40	2.45	6.13	3.42	3.16
**Median**	15.00	15.50	21.50	16.50	13.50
**Min**	8.50	11.00	11.50	10.50	7.50
**Max**	20.50	22.00	36.50	25.00	21.00

**Table 2:** ANOVA post-hoc test with Bonferroni correction between intraocular pressure evaluations (in mmHg) during different procedure times. The mean difference is significant at level <0.05.

				95% Confidence Interval	
		Mean Difference	*p*	Lower Limit	Upper Limit
**Sitting position**	**Left lateral decubitus position**	−0.50	1.000	−3.5487	2.5487
	**D2**	−6.40	**0.000**	−9.4487	−3.3513
	**End of procedure**	−1.22	1.000	−4.2687	1.8287
	**10 min later**	1.84	0.869	−1.2087	4.8887
**Left lateral decubitus position**	**Sitting position**	0.50	1.000	−2.5487	3.5487
	**D2**	−5.90	**0.000**	−8.9487	−2.8513
	**End of procedure**	−0.72	1.000	−3.7687	2.3287
	**10 min later**	2.34	0.301	−0.7087	5.3887
**D2**	**Sitting position**	6.40	**0.000**	3.3513	9.4487
	**Left lateral decubitus position**	5.90	**0.000**	2.8513	8.9487
	**End of procedure**	5.18	**0.000**	2.1313	8.2287
	**10 min later**	8.24	**0.000**	5.1913	11.2887
**End of procedure**	**Sitting position**	1.22	1.000	−1.8287	4.2687
	**Left lateral decubitus position**	0.72	1.000	−2.3287	3.7687
	**D2**	−5.18	**0.000**	−8.2287	−2.1313
	**10 min later**	3.06	0.048	0.0113	6.1087
**10 min later**	**Sitting position**	−1.84	0.869	−4.8887	1.2087
	**Left lateral decubitus position**	−2.34	0.301	−5.3887	0.7087
	**D2**	−8.24	**0.000**	−11.2887	−5.1913
	**End of procedure**	−3.06	0.048	−6.1087	−0.0113

## Data Availability

The datasets generated and analyzed during the current study are available from the corresponding author on reasonable request.
